# Practices Regarding COVID-19 among Online Respondents in Nepal: A Descriptive Cross-sectional Study

**DOI:** 10.31729/jnma.6428

**Published:** 2021-05-31

**Authors:** Sudeep Chapagain, Aastha Singh, Prabina Basnet, Rasik Neupane, Nirdesh Pokhrel

**Affiliations:** 1 School of Medicine, Patan Academy of Health Sciences, Lagankhel, Lalitpur, Nepal; 2 Department of Social Sciences, Tribhuvan University, Kirtipur, Kathmandu, Nepal; 3 Kathmandu Hospital, Tripureshwor, Kathmandu, Nepal; 4 Junior Doctors Committee, Nepal Medical Association, Bagbazar, Nepal

**Keywords:** *COVID-19*, *pandemic*, *practices*

## Abstract

**Introduction:**

The alarming rate of rise in COVID-19 cases led to lockdown in Nepal in order to curb the transmission and spread of the virus among the general public. This research was conducted to know the practices regarding the COVID-19 among the general population of Nepal. The aim of the study is to find out the value of different practices regarding COVID-19 which includes use of face masks, avoiding crowded places and hand hygiene.

**Methods:**

This was a descriptive cross-sectional study conducted among 509 online respondents residing 28th November to 15th December 2020 in Nepal. Ethical approval for the study was taken from the Nepal Health Research Council (Reference number 1350). Convenient sampling method was used. Data was analyzed using Microsoft Excel and Software Statistical Package for Social Sciences version 26.0. Point estimate at 95% Confidence Interval was calculated along with frequency and proportion for binary data.

**Results:**

Out of 509 participants, 492 (96.66%) (Confidence Interval= 95.1%-98.2%) uses mask, 437 (85.85%) (Confidence Interval= 82.82%-88.87%) avoided going to crowded places and 503 (98.82%) (95% Confidence Interval= 97.88%-99.76%) practiced hand hygiene during lockdown. Among 492 (96.66%) who wore face mask during the lockdown, 482 (94.69%) were still continuing using the face mask after the restriction was eased.

**Conclusions:**

The study concludes good practises regarding COVID-19 among Nepalese population but is still not satisfying. This depicted adequate awareness among the public as a result of adequate dissemination of information and resources during the lockdown.

## INTRODUCTION

The World Health Organization (WHO) first learned of this new virus on 31st December 2019, following a report of a cluster of cases of ‘viral pneumonia’ in Wuhan, People's Republic of China.^1,2^ The first case of COVID-19 in Nepal was confirmed by Ministry of Health and Population (MoHP) on 23rd January 2020^3^ So, public health measures and practices became a key aspect to control the pandemic.

As the cases of the COVID-19 was rising in Nepal despite the government's effort, containment and slowing the transmission was possible only through following the public health guidelines and adherence to effective rules like using masks, frequent handwashing, maintaining physical distance and avoidance of the crowded places.^4^

The aim of the study is to find out the value of different practices regarding COVID-19 which includes use of face masks, avoiding crowded places and hand hygiene. Therefore, the study will give an idea about the present scenario and evidence-based information useful to policy makers and practitioners involved in controlling COVID-19.

## METHODS

A descriptive cross-sectional survey was conducted from 28th November to 15th December 2020 among online respondents residing in Nepal. Ethical approval for the study was taken from the Nepal Health Research Council (NHRC) ( Reference no. 1350). Every Nepalese citizen residing in Nepal above 18 yrs of age who use the internet and social media and who can understand and read Nepali or English Language are included in this study. Respondents who did not fill up the form completely were excluded from the study. Convenient sampling method was used. Informed consent was taken from each participant which was attached in the google form itself. Minimum sample size was calculated using the formula,


$$\begin{array}{ll} \text{n} & \text{= }{\text{Z}}^{\text{2}}\times \text{p}\times \text{q }/{\text{e}}^{\text{2}}\\ & \text{= }{\left(1.96\right)}^{\text{2}}\times {\left(0.5\right)}^{2}\times (1-0.5)/{(\text{0.05)}}^{\text{2}}\\ & \text{= }385 \end{array}$$

Where,

n = minimum required sample size
Z = Confidence Interval at 95%
p = prevalence taken 50% for maximum sample size
q = 1-p
e = margin of error, 5%

Data was collected using the previously validated questionnaire with some modifications using the Google forms by maintaining anonymity of the respondents. Questionnaire was divided into two sections: 1) Demographic and 2) Practice questions. A pilot study was conducted to validate the modified questionnaire. Point estimate at 95% Confidence Interval was calculated along with frequency and proportion for binary data. Data was analyzed using Microsoft Excel and Software Statistical Package for Social Sciences (SPSS) version 26.0.

## RESULTS

Public practices regarding COVID-19 were assessed by using three main questions. The questions investigated 1) avoidance of crowded places, 2) use of face masks and 3) practice of hand hygiene after the lockdown was placed by the government of Nepal. After the initiation of lockdown majority of respondents 437 (85.85%) (at 95% Confidence Interval= 82.82-88.87) were avoiding crowded places and few of 72 (14.14%) were still reluctant to do so. Still a large number of respondents 361 (70.92%) kept avoiding crowded places whereas 148 (29.08%) failed to do so after the restrictions were removed. Majority of the participants 492 (96.66%) (at 95% Confidence Interval= 95.1%-98.2%) wore face mask and very few of them 17 (3.34%) were not wearing face mask after the lockdown was imposed and greater number of respondents 482 (94.69%) kept wearing face mask whereas 27 (5.3%) of them failed to continue the use of masks after the release of restriction placed by the government. Similarly, it was found that a large number of people 503 (98.82%) (at 95% Confidence Interval= 97.88%-99.76%) practiced proper hand hygiene and only 6 (1.17%) were not following it. While, vast majority of respondents 490 (96.26%) kept following the practice whereas only 19 (3.73%) failed to do so soon after the government lifted the restrictions (Table 1).

**Table 1:** Practice pattern during COVID-19 among online respondents (n = 509).

		Wearing Face Mask n (%)		Avoiding Crowd n (%)		Hand Hygiene n (%)	
		No	Yes	No	Yes	No	
**Gender**	Male	261 (96)	11 (4)	231 (84.90)	41 (15.10)	266 (97.80)	6 (2.20)
	Female	231 (97.50)	6 (2.50)	206 (86.90)	31 (13.10)	237 (100)	0 (0)
**Age Group**	<20	89 (100)	0 (0)	71 (79.80)	18 (20.20)	89 (100)	0 (0)
	21-30	294 (95.80)	13 (4.20)	268 (87.30)	39 (12.70)	301 (98)	6 (2)
	31-40	68 (98.60)	1 (1.40)	58 (84.10)	11(15.90)	69 (100)	0 (0)
	>40	41 (93.20)	3 (6.80)	40 (90.90)	4 (9.16)	44 (100)	0 (0)
**Residing Area**	Metropolitan/Sub-Metropolitan	223 (88.10)	30 (11.90)	223 (88.10	30 (11.90)	249 (98.40)	4 (1.60)
	Municipality	200 (84)	38 (16)	200 (84)	38 (16)	236 (99.20)	2 (0.80)
	Rural Municipality	14 (77.80)	4 (22.20)	14 (77.80)	4 (22.20)	18 (100)	0 (0)
**Occupation**	Student	236 (95.90)	10 (4.10)	208 (84.6)	38 (15.40)	242 (98.40)	4 (1.60)
	Unemployed	21 (100)	0 (0)	17 (81)	4 (19)	21 (100)	0 (0)
	Retired	1 (100)	0 (0)	1 (100)	0 (0)	1 (100)	0 (0)
	Private Sector Employee	102 (97.10)	3 (2.90)	96 (91.40)	9 (8.60)	104 (99)	1 (1)
	Public/Government Sector Employee	39 (92.90)	3 (7.10)	40 (95.20)	2 (4.80)	42 (100)	0 (0)
	Self employed	25 (100)	0 (0)	17 (68)	8 (32)	24 (96)	1 (4)
	Health Worker	60 (98.40)	1 (1.60)	50 (82)	11 (18)	61 (100)	0 (0)
	Other	8 (100)	0 (0)	8 (100)	0 (0)*	8 (100)	0 (0)
**Education**	Below SLC/SEE	1 (100)	0 (0)	1 (100)	0 (0)	1 (100)	0 (0)
	SLC or SEE	6 (100)	0 (0)	5 (83.30)	1 (16.70)	5 (83.30)	1 (16.70)
	+2 level	63 (98.40)	1 (1.60)	52 (81.30)	12 (18.80)	64 (100)	0 (0)
	Undergraduate	237 (96)	10 (4)	210 (85)	37 (15)	244 (98.80)	3 (1.20)
	Graduate and above	185 (96.90)	6 (3.10)	169 (88.50)	22 (11.50)	189 (99)	2 (1)*

Other seven relevant questions of practice were also assessed (Table 2).

**Table 2:** Practice specific questions.

Practice Questions	Response	n (%)
Which type of mask do you use?	Normal surgical mask	345 (67.79)
	Medical mask (N95, N99, FFP2)	151 (29.66)
	Non- medical masks (Homemade or fabric mask)	12 (2.35)
	Do not use mask	1 (0.10)
For how long do you use a single mask?	One time use	283 (55.59)
	Multiple use without washing	97 (19.05)
	Multiple use after washing	129 (25.34)
For how long do you use a single mask?	In dustbin with other wastes	307 (60.13)
	Dispose separately	159 (31.23)
	Dispose anywhere feasible	43 (8.44)
What do you prefer to do?	Handwashing with soap water/ sanitizer	485 (95.28)
	Using gloves	24 (4.71)
What do you prefer to wash your hands with?	Soap water	213 (41.86)
	Alcohol based sanitizer	22 (4.32)
	Both	274 (53.83)
For how long do you wash your hands?	10-20 seconds	180 (35.36)
	20-30 seconds	200 (39.29)
	30-40 seconds	74 (14.53)
	40-60 seconds	25 (4.91)
	More than 1 minute	30 (5.89)
What is the minimal social distance that you maintain?	Less than 1 meter	100 (19.64)
	Around 1-3 meter	360 (70.72)
	Around 3-6 meter	40 (7.85)
	More than 6 meter	9 (1.76)

The study included 509 participants out of which more than half were female 272 (53.4%) and others were male 237 (46.6%). Among the total participants, 368 (72%) of them were of age group 18-29 years and 125 (25%) were of age group 30-49 years. Whereas, only 16 (3%) of the respondents were of age group 50 years and above (Table 3).

**Table 3:** Demographic Characteristics of the Respondents.

Characteristics		n (%)
Gender	Male	237 (46.6)
	Female	271 (53.4)
Age	18-29	368 (72.29)
	30-49	125 (24.55)
	Above 50	16 (3.14)
Residency Area	Metropolitan/ Sub-Metropolitan	253 (49.7)
	Municipality	238 (46.8)
	Rural Municipality	18 (3.5)
Occupation	Student	246 (48.3)
	Unemployed	21 (4.1)
	Retired	1 (0.2)
	Private sector employee	105 (20.6)
	Public/ Government employee	42 (8.3)
	Self-employed	25 (4.9)
	Health worker	61 (12)
	Other	8 (1.6)
Education	Below SLC/SEE	1 (0.2)
	SLC/SEE	6(1.2)
	+2 Level	64 (12.6)
	Undergraduate	247 (48.5)
	Graduate and above	191 (37.5)

Descriptive analysis of other relevant practice questions related to mask use, hand hygiene and social distancing were done. Most of the respondents showed good practice by using normal surgical and medical masks, following proper hand hygiene and maintaining adequate social distancing.

## DISCUSSION

This study was designed to elicit the practices regarding the COVID-19 in Nepal after lockdown was done by the government among various population sub-groups. There has been a significant improvement in practices regarding COVID-19 when the restrictions were placed. 85.8% of respondents avoided going to crowded places during lockdown and 70.9% still followed the practice after the restriction was eased. This avoidance of crowded places might be because of the prohibition by the government to take part in activities involving the crowds like marriage, religious activities, travelling etc. and also increasing awareness among the people during the lockdown. The decrease after the ease of restrictions could be attributed to lifting of the prohibition to travel and other activities. In case of hand hygiene 98.8% of the respondents were practicing hand hygiene during lockdown and majority of them 96.2% continued following hand hygiene thereafter when the government lifted some restrictions. Similarly, the number of respondents using face masks were 96.6% during lockdown and it remained 94.69% when the restrictions were decreased.

Furthermore, this study also showed that participants from rural municipalities were more likely to go to crowded places compared to the participants from municipalities and metropolitans which could be due the strict lockdown in the cities than in the villages. It can also be due to the higher knowledge about the disease prevention in participants from the city areas. Majority of the participants (345) used a normal surgical mask, out of which 283 preferred single use of the mask. 485 preferred handwashing rather than using gloves, out of which 213 preferred soap and 274 preferred both hand sanitizer and soap for washing hands. These practice findings showed that there was enough availability and accessibility of the mask, soaps and hand sanitizers as well as awareness regarding the methods of prevention of the COVID-19 spread.

Similar online cross-sectional study conducted in 766 Nepalese residents during lockdown revealed that 94.9% of participants had not been to the crowded places recently, 88.2% were wearing masks correctly and the majority of the participants i.e 85.7% mentioned they were following hand hygiene while in our study 85.8% had not been to the crowded places, 96.6% wore masks while going outside and 98.8% followed hand hygiene during lockdown.^5^ These findings related to practices were similar to the findings after lockdown in our study.

A previous study conducted in Malaysia^6^ showed that the 83.4% of participants were avoiding crowded places, almost half of the participants i.e. 51.2% reported wearing a face mask when going out in public and majority of participants i.e 87.8% reported that they have practiced proper hand hygiene by frequently washing their hands. While in our study 85% had not been to crowded places, 96.6% wore masks while going outside and 98.8% followed hand hygiene during lockdown. This showed that there was better practice of wearing face masks while going to crowded places in Nepal than in Malaysia. The availability of face masks to the general people and the timely awareness raised regarding the use of face masks by the media as well as health workers could have led to the more use of masks in Nepal. In Malaysian study, it was reported that there was scarcity of masks and also the government had focused more on the mask use only by the COVID-19 positive individuals.

The findings of a similar study conducted in China by Zhong, et al.^7^ showed that 96.4% of the respondents avoided the crowded places whereas 98.0% used the face masks while leaving their home during the lockdown. These findings were similar to our study.

Our study revealed improvement in practices after the lockdown was started, which signifies adequate dissemination of information regarding COVID-19 to the general public. It also reassures them about the enough availability and distribution of resources to fight against the pandemic like face masks, hand sanitizers among the public. The improvements in practices after the lockdown might have played a major role in controlling the spread of the virus. This exhibits the need for continuous effort of government and local authorities' strategies and activities to fight against this pandemic and help decrease the impact on every aspect of society (e.g. education, finance, health, supply of basic needs). As the lockdown has again been imposed, good practices should be continued in order to control the transmission in the future as well. In order to ensure this, the government should work together with the general public through coordination with the local government and authorities. This joint effort can help Nepal win the battle against the pandemic in the near future.

## CONCLUSIONS

The practice pattern is good but still not satisfying. This depicted adequate awareness among the public as a result of adequate dissemination of information and resources during the lockdown. This might have played a major role in controlling the spread of the virus and decreasing in the number of positive cases. These good practices are the key to controlling transmission in the future as well. Government and local authorities should work hand in hand with the general public to continue such practices along with an increase in awareness about these practices to the root level of the community. Underprivileged population can benefit from the programs targeted to their community such as distribution of masks and soap, demonstration of hand washing technique in schools and every household. With these joint efforts between government and public only, Nepal can win the battle against the COVID-19.

## References

[1] She J, Jiang J, Ye L, Hu L, Bai C, Song Y (2020). 2019 novel coronavirus of pneumonia in Wuhan, China: emerging attack and management strategies. Clin Transl Med.

[2] World Health Organization. (2020). World Health Organization (WHO) Coronavirus disease (COVID-19) Question and Answers [Internet].

[3] Ministry of Health and Population: MOHP Nepal (2020). COVID-19 Situation Update Report 2020 [Internet].

[4] World Health Organization (2020). World Health Organization (WHO) Coronavirus disease (COVID-19) advice for the public [Internet].

[5] Paudel S, Shrestha P, Karmacharya I, Pathak OP (2020). Knowledge, attitude and practices (KAP) towards COVID-19 among Nepalese Residents during COVID-19 outbreak: An online cross-sectional study. BMC Public Health.

[6] Azlan AA, Hamzah MR, Sern TJ, Ayub SH, Mohamad E (2020). Public 7. knowledge, attitudes and practices towards COVID-19: A cross-sectional study in Malaysia. PLoS One.

[7] Zhong BL, Luo W, Li HM, Zhang QQ, Liu XG, Li WT, Li Y (2020). Knowledge, attitudes, and practices towards COVID-19 among Chinese residents during the rapid rise period of the COVID-19 outbreak: a quick online cross-sectional survey. Int J Biol Sci.

